# A novel TLR4 binding protein, 40S ribosomal protein S3, has potential utility as an adjuvant in a dendritic cell-based vaccine

**DOI:** 10.1186/s40425-019-0539-7

**Published:** 2019-02-28

**Authors:** Hyun Jin Park, Gun-Young Jang, Young Seob Kim, Jung Hwa Park, Sung Eun Lee, Manh-Cuong Vo, Je-Jung Lee, Hee Dong Han, In Duk Jung, Tae Heung Kang, Yeong-Min Park

**Affiliations:** 1 Department of Immunology, KU Open Innovation Center, School of Medicine, Konkuk University, 268, Chungwondaero, Chungju, 274798 South Korea; 2 Hematology-Oncology, Chonnam National University Hwasun Hospital, Hwasun-gun, Jeollanam-do 58128 South Korea

**Keywords:** Dendritic cell (DC), Toll-like receptor 4 (TLR4), DC based vaccine, 40S ribosomal protein S3 (RPS3)

## Abstract

**Background:**

Dendritic cells (DCs) are professional antigen presenting cells (APCs), which can activate antigen-specific CD8+ T cell immunity, resulting in tumor clearance. Immature DCs are usually stimulated by various adjuvants through their immune receptors. Among them, Toll-like receptor 4 (TLR4) has an important role in activating DCs to cause their maturation. In fact, TLR4 is well-known to induce innate and adaptive immune responses against various external microbial or internal damage associated molecular patterns (DAMP). LPS is widely regarded as a strong stimulator of TLR4 signaling. However, LPS is inappropriate for use in humans since it is an endotoxin. Unfortunately, other TLR4 ligands such as HMGB1 or heat shock proteins have weak adjuvant effects. Therefore, there is a need to identify novel, biocompatible, strong, TLR4 ligands.

**Methods:**

40S ribosomal protein S3 (RPS3) was screened through pull-down assay using TLR4. BMDCs from wild type (WT) and TLR4 knock-out mice were treated by RPS3 to identify the activation and maturation of DCs. T cell generation including memory T cells, tumor prevention, and treatment experiments were performed with BMDCs based vaccination. Also, human DCs originated from patients were treated by RPS3 to confirm the activation and maturation of DCs.

**Results:**

In this study, we identified 40S ribosomal protein S3 (RPS3) through a pull-down assay using a variety of human cancer cell-derived proteins that could bind to TLR4. RPS3 was released from tumor cells following treatment with an anticancer drug, and it was shown that the released RPS3 binds to TLR4. Recombinant RPS3 induced maturation and activation of DCs, and following pulsing with tumor specific antigens, these DCs could be used as a vaccine to significantly increase tumor specific CD8^+^IFN-γ^+^ T cells, and provide both tumor prevention and tumor treatment effects. The effect of RPS3 on DC maturation and its utility as a vaccine were shown to be dependent on TLR4 using TLR4 knockout mice.

**Conclusions:**

This study therefore proved that human cancer cell-derived RPS3, a novel TLR4 ligand, has great potential as an adjuvant in tumor-specific antigen DC-based vaccines.

**Electronic supplementary material:**

The online version of this article (10.1186/s40425-019-0539-7) contains supplementary material, which is available to authorized users.

## Background

Pattern recognition receptors (PRRs) are well known to function in the early stages of the innate immune response and recognize pathogen-associated molecular pattern (PAMPs) from external microorganisms or damage-associated molecular patterns (DAMPs) from damaged tissues [1]. There are four types of PRRs, including C-type lectin receptors (CLRs), RIG I-like receptors (RLRs), NOD-like receptors (NLRs), and Toll-like receptors (TLRs) [2–4]. TLRs recognize various ligands (e.g. DNA, RNA, proteins), and activate intracellular signaling pathways (e.g., MAPK, NF-кB) and produce pro-inflammatory cytokines in antigen presenting cells (APCs), e.g., dendritic cell (DCs) [5–7]. Among the TLRs, TLR4 is the mostly well-studied, and it was the first TLR found in humans that can recognize lipopolysaccharide (LPS), a component present in gram-negative bacteria [8]. Its ligands also include HMGB1, heat shock proteins, fusion proteins, as well as other proteins [2].

DCs express TLRs to which numerous ligands can bind, thereby inducing the expression of co-stimulatory molecules and the secretion of pro-inflammatory cytokines [9]. DCs have been shown to be important not only in the innate immune response, but also in the adaptive immune response where mature DCs can present uptaken antigen to naïve T cells leading to their activation [10, 11]. DC-based cancer vaccines have been produced, and of the different cancer vaccines available for use in humans (antigen/adjuvant vaccines, anti-idiotype vaccines, DNA vaccines, tumor cell vaccines) are the most preferred [12–14]. As an example, Provenge, a DC-based cancer vaccine drug, is the only such vaccine approved by the FDA [15, 16]. In order to generate DC-based cancer vaccines, an adjuvant that binds to TLRs that can induce maturation and activation of DCs is needed. It is well known that LPS, a TLR4 ligand, has a significant effect on inducing maturation and activation of DCs through the MyD88 and TRIF cell signaling pathways [17]. However, LPS is not appropriate for use in humans because it is an endotoxin, which is derived from the cell walls of gram-negative bacteria. Instead, the TLR9 ligand CpG, or the TLR3 ligand I:C, are commonly used, but require a large amount of CpG or I:C to induce DC maturation and activation. Therefore, we need to find a new adjuvant that can bind to TLR4 to effectively induce DC maturation.

To identify an efficient adjuvant that binds to TLR4, intracellular proteins in human tumor cells were screened through a pull-down assay. About twenty TLR4-binding proteins were found, half of which were ribosomal protein families. These ribosomal proteins were purified and tested for their ability to induce maturation and activation of DCs. Among the ribosomal proteins studied, 40S ribosomal protein S3 (RPS3) was identified as a possible adjuvant for use in DC-based vaccines.

RPS3 is a component of the 40S/60S ribosome. RPS3 is involved in the regulation of apoptotic signaling pathways and the control of gene expression [18, 19]. Specifically, it has been shown that DNA damage is repaired and that NF-κB driven gene expression is activated after binding to the KH domain, the DNA binding site, that resides at the N-terminal of RPS3 (a.a. 21–92). Moreover, RPS3 has also been shown to bind to Hsp70, Hsp90, several enzymes, and kinases, as well as other proteins [20–27]. In addition to the many intracellular functions of RPS3, it has been shown that RPS3 is secreted after its N-linked glycosylation, although how it acts extracellularly is not known [28].

In the present study, we screened RPS3 from human tumor cells as a new TLR4 ligand. We examined the effects of RPS3 on maturation and activation of mouse and human dendritic cells in vitro. Furthermore, DC vaccination with RPS3 and tumor cell specific peptides confirmed the utility of RPS3 as an adjuvant in vivo. In addition, we showed that the adjuvant effects of RPS3 were dependent on TLR4.

## Methods

### Mice

Female C57BL/6 mice, 6–8 weeks of age, were purchased from Orient Bio (Seongnam, Korea). TLR4 knockout mice (C57BL/10ScNF) were purchased from Jackson Laboratories (Maine, USA). All mice were kept under specific pathogen-free conditions in accordance with the animal care guidelines approved by the Institutional Animal Care and Use Committee (IACUC) of Konkuk University.

### Cells

TC-1 is a transformed mouse lung epithelial cell expressing the HPV-16 E7 gene [29]. The human cell lines CFPAC-1, SNU17, and SKOV3, as well as the mouse lymphoma EG.7 cell line (mouse EL4 cells transfected with the OVA cDNA) were obtained from ATCC (Virginia, USA). The mouse melanoma cancer cell line B16F10 was obtained from the Korean Cell Line Bank. Cells were incubated at 37 °C in an atmosphere of 5% CO_2_ and cultured in RPMI-1640, DMEM, or IMEM (Biowest, Rue de la Caille, France) medium supplemented with 10% fetal bovine serum (Biowest, Rue de la Caille, France) and 50 U/mL penicillin streptomycin (Biowest, Rue de la Caille, France). HEK293 cells expressing hTLR4-MD2-CD14 were cultured according to the protocol provided by InvivoGen (CA, USA). Human monocytic THP-1 cells were differentiated by treating with PMA (200 μg/mL) (Sigma, Missouri, USA) [30]. Mouse dendritic cells were isolated from mice bone marrow and cultured in RPMI-1640 medium supplemented with 10% fetal bovine serum, 50 U/mL penicillin streptomycin, 1000 μM 2-mercaptomethanol (Gibco, MA, USA), recombinant mouse IL-4, and granulocyte-macrophage colony-stimulating factor (JW CreaGene, Seongnam, Korea). The culture medium was changed every two days and the cells were differentiated for six days. After DC differentiation, CD11c + DCs were used in our experiments after gating through Flow Cytometry. Human DCs were differentiated after the isolation of monocytes from peripheral blood mononuclear cells (PBMCs). And due to the excess of number of characters, the detailed protocols are described in supplementary data (Additional file 1).

### Purification of recombinant RPS3 protein

The RPS3 expression vector was constructed from a cDNA derived from the human pancreatic adenocarcinoma CFPAC-1 cell line. The insert DNA along with cloning sites (NdeI and NotI) was synthesized using a CFPAC-1 cDNA and the RPS3 primer (forward primer; 5′-CATATG GCAGTGCAAATATCCAAGAAG-3′, reverse primer; 5′-GCGGCCGC TTATGCTGTGGGGACTGG-3′). This DNA was then inserted into the pET28b bacterial expression vector. Two fragments of the recombinant RPS3 protein (1–95 and 91–243) were synthesized using the 95-amino acid reverse primer (5′-GCGGCCGC TTAACCTCTAGTGGCCACCTT-3′) and the 91-amino acid forward primer (5′-CATATG GTGGCCACTAGAGGTCTGTGT-3′). The detailed protocols are described in supplementary data (Additional file 1).

### Luciferase assay

Luciferase activity was assessed using the Dual-Glo Luciferase Assay System (Promega, Wisconsin, USA). The pGL4.32[luc2P/NF-κB-RE/Hygro] vector (Promega) and the pRL-TK vector (Promega) were transfected into 293/hTLR4A-MD2-CD14 cells to assess the activation of NF-κB activity by the RPS3 protein. Cells (2 × 10^4^/well were seeded into a 96-well white plate (SPL, Pocheon, Korea) and then transfected with each of the two plasmids. Cells were then incubated at 37 °C, after 16 h, RPS3 (1 μg/mL), GFP (5 μg/mL), or LPS (100 ng/mL) were added to the culture and the incubation was continued for a further 2 h. Firefly luminescence, as a measure of NF-κB activity value and Renilla luminescence as a normalization value, were measured according to the protocol provided by the assay kit manufacturer (Promega). Luciferase activity was expressed as the ratio of firefly luminescence:Renilla luminescence for each well.

### Enzyme-linked immunosorbent assay (ELISA)

The levels of secreted cytokines derived from mouse BMDCs and THP-1 cells were measured using ELISA kits and followed the individual ELISA kit’s manufacturer’s instructions for mouse and human TNF-α, IL-1β, IL-6, IL-10, IL-12p70 (ebioscience, MA, USA) and IFN-β (pbl). The levels of cytokines (IL-10 and IL-12p70) secreted from human DCs were determined using ELISA kits obtained from BD Bioscience (New Jersey, Korea).

### Flow cytometry analysis

Mouse BMDCs, or THP-1 cells, were stained with the following surface markers; FITC-CD11c, PE-CD40, PE-CD80, PE-86, PE-MHC-I, PE-CD83, or PE-CCR7 (BioLegend). Human DCs were stained with FITC-labeled anti-HLA-DR, PE-labeled anti-CD80, and FITC-labeled anti-CD86 (Miltenyi). Cell activation was analyzed using a FACSCalibur with CELLQuest (Becton Dickinson, New Jersey, USA). To assess CD8^+^ T cell generation in vivo, splenocytes from treated or vaccinated mice were stained with PE-labeled anti-CD8a (BioLegend) and FITC-labeled anti-IFN-γ (BioLegend). FITC-labeled IFN-γ staining was performed using an intracellular staining kit (BD Bioscience).

### TLR4 blocking antibody

MAb mTLR4/MD2 (InvivoGen) was pre-treated on mouse BMDCs 1 h before the treatment of RPS3 of LPS.

### Western blotting

Mouse BMDCs were treated with RPS3 (1 μg/mL) over a time course (0, 10, 20, 30, 40, 50, and 60 min) and the cells harvested. Cells were lysed using RIPA buffer (0.5% NP-40, 1 mM EDTA, 50 mM Tris-Cl pH 8.0, 120 mM NaCl, protease inhibitor cocktail, 100 mM PMSF, 0.5 M NaF) on ice for 1 h. The detailed protocols are described in supplementary data (Additional file 1).

### Immunoprecipitation

His-tagged human recombinant TLR4 was bound to the Ni-NTA resin at 4 °C using a binding buffer of 1× PBS (pH 7.4). After 12 h, the TLR4-Ni-NTA beads were washed five times with PBS, and then the B16F10 tumor cell supernatant was added and the incubation continued for 12 h at 4 °C. The B16F10 tumor cells were treated with 10 μg/mL of doxorubicin. After 2 h, the media was changed to Opti-MEM (Gibco, MA, USA). Two days later, 500 μL of the supernatant was collected and concentrated to 100 μL using a Centricon filter (MERK, Darmstadt, Germany). Following this the beads were washed five times with PBS. Residual buffer was then removed from the resin, and 50 μL of 5 × SDS loading buffer was then added and the beads were boiled. The eluted proteins were separated by electrophoresis on 10% SDS-PAGE gels and the separated protein transferred to a PVDF membrane.

### In vivo experiments

Tumor antigen-specific CD8^+^IFN-γ^+^ T cell generation, tumor prevention, and treatment experiments were performed using mice injected with different cell formulations: no vaccination, PBS-treated immature DCs, immature-DCs loaded with tumor antigen peptide, RPS3-treated mature DCs, RPS3-treated mature DCs loaded with tumor antigen peptide, and LPS-treated mature DCs loaded with tumor antigen peptide. Mice were vaccinated with BMDCs (2 × 10^6) on the footpad twice at intervals of one week. The tumor-specific antigen peptides OVA (SIINFEKL) and E7 (RAHYNIVTE) (Anygen, Gwangju, Korea) were used. Tumor antigen-specific CD8^+^IFN-γ^+^ T cell generation in the mouse spleens was measured using flow cytometry.

The tumor prevention experiment was performed by subcutaneous injection of mouse cancer cells (5 × 10^6^ EG.7 cells, 2.5× 10^5^ TC-1 cells) seven days after the last DC injection.

In the tumor treatment experiments, cancer cells (1 × 10^6^ EG.7, 2 × 10^5^ TC-1) were injected subcutaneously. After five days, BMDCs were injected and the tumor size was measured every 2 to 3 days. The size of the tumor mass was calculated using the formula (length × width × width × 1/2). Measurement of tumor size and mouse survival was performed until the tumor diameter was over 2 cm or the mice were dead.

Mice were vaccinated with RPS3-treated BMDCs pulsed with OVA twice at intervals of one week. The memory T cell experiment was performed using the following injection groups: no vaccination with either tumor injection or not, and vaccination with either tumor injection or not. Seven weeks from the last BMDC injection, 1 × 10^6^ EG.7 tumor cells were injected subcutaneously. Seven days later, CD8^+^IFN-γ^+^ T cells generation in the spleen was measured by flow cytometry and the mice were observed until the tumor diameter was over 3 mm.

(A) Identification of intracellular RPS3 in human and mouse tumor cells by western blot. (B) Human and mouse tumor cells were treated with doxorubicin (10 μg/mL). After 2 h, the media was changed to Opti-MEM media and the cells were incubated overnight. Release of RPS3 into the culture supernatant was assessed by western blot. (C) After doxorubicin treatment (10 μg/mL) of B16F10 cells, the binding of released RPS3 in the culture supernatant and recombinant TLR4 was confirmed by immunoprecipitation. (D) Purification of recombinant RPS3 from *E. coli* assessed by Coomassie Brilliant Blue (CBB) staining and western blotting. (E) TLR4-MD2 expressing HEK293 cells were treated with recombinant RPS3 (0.01, 0.1, 1 μg/ml), GFP (5 μg/mL) or LPS (100 ng/mL) and NF-кB activity was measured by luciferase assay (**; *p* < 0.01). (F) The binding affinity between recombinant RPS3 (1 μg) or LPS (1μg) and TLR4 (1 μg) was assessed using BLITz. All the experiments were performed three times.

Mouse BMDCs were treated with RPS3 (0.01, 0.1, or 1 μg/mL), GFP (5 μg/mL), or LPS (100 ng/mL) and (A) pro-inflammatory cytokines (TNF-α, IL-1β, IL-6, IL-10, and IL-12p70), as well as IFN-β secretion levels, in the cell culture supernatant were measured by ELISA (*; *p* < 0.05, **; p < 0.01 and ***; *p* < 0.001). (B) The expression of co-stimulatory molecules (CD40, CD80, CD86, and MHC-I) in CD11c + BMDCs were assessed by flow cytometry. (C) Mouse BMDCs were treated with RPS3 protein (1 μg/mL) for different periods of time (0, 10, 20, 30, 40, 50, or 60 min) and activation of the MAPK (ERK, P38, and JNK), AKT and NF-κB cell signaling pathways were analyzed by western blot. All the experiments were performed three times.

All cells were treated with RPS3 (0.1, 1 μg/mL), GFP (5 μg/mL) or LPS (100 ng/mL). (A) The expression levels of co-stimulatory molecules (CD80, CD83, and CD86) and CCR7 in human THP-1 cells were analyzed by flow cytometry. (B) The levels of cytokines (TNF-α, IL-6, IL-10, and IFN-β) secreted from human THP-1 cells were measured by ELISA. (C) The expression of co-stimulatory molecules (HLA-DR, CD80, and CD86) in human DCs were analyzed. (D) The levels of cytokines (IL-10 and IL-12p70) secreted from human DCs were measured. All the experiments were performed three times. (*; *p* < 0.05, **; *p* < 0.01 and ***; *p* < 0.001).

(A, B) Mouse BMDCs were separated into the following groups; no vaccination, PBS-treated immature DCs, immature DCs loaded with tumor antigen peptide, RPS3-treated mature DCs, RPS3-treated mature DCs loaded with tumor antigen peptide, and LPS-treated mature DCs loaded with tumor antigen peptide. DCs were treated with RPS3 (1 μg/mL) or LPS (100 ng/mL) and then pulsed with OVA (A) and/or E7 (B) tumor specific antigen peptides. The treated DCs were then injected into the footpads of mice. After 1 week the last injection of BMDCs, the spleen was isolated and the number of tumor specific CD8^+^IFN-γ^+^ T cells produced was counted by Flow Cytometry (C, D). After vaccination by the different DCs, EG.7 (C) or TC-1 (D) cells were subcutaneously injected and tumor formation was observed for 60 days. All the experiments were performed three times. IBM SPSS Statistics Base 22.0 was used as statistical method to analyze differences between experimental groups. (**; *p* < 0.01 and ***; *p* < 0.001)

(A, B) EG.7 (A) or TC-1 (B) tumor cells were injected subcutaneously into mice. Following this, mouse BMDCs were separated into the following groups: no vaccination, PBS-treated immature DCs, immature DCs loaded with tumor antigen peptide, RPS3-treated mature DCs, RPS3-treated mature DCs loaded with tumor antigen peptide, and LPS-treated mature DCs loaded with tumor antigen peptide. Five days after tumor injection, the tumor size was measured and mouse survival was observed until the tumor diameter was over 2 cm or the mouse had died. (C, D) Mouse BMDCs were separated into the following groups: no vaccination with either tumor injection or not and vaccination with either tumor injection or not. Seven weeks after the last BMDC injection, EG.7 tumor cells were injected subcutaneously into mice. The spleen was isolated from mice and the number of tumor-specific memory T cells produced was determined by Flow Cytometry (C) and tumor formation was observed (D). All the experiments were performed three times. IBM SPSS Statistics Base 22.0 was used as statistical method to analyze differences between experimental groups. (**; *p* < 0.01 and ***; *p* < 0.001)

(A, B) WT and TLR4−/− mouse BMDCs were treated with RPS3 (1 μg/mL), GFP (5 μg/mL), or LPS (100 ng/mL) and the levels of secreted cytokines (TNF-α, IL-1β, IL-6, IL-10, IL-12p70, and IFN-β) as well as the expression of co-stimulatory molecules (CD40, CD80, CD86, and MHC-I) were measured. (C) RPS3 (1 μg/mL) was used to treat WT and TLR4−/− mouse BMDCs for different periods of time (0, 10, 20, 30, 40, 50, and 60 min) and the activation of the MAPK (ERK, P38 and JNK), AKT, and NF-κB cell signaling pathways were analyzed by western blot. Total MAPK and AKT levels were used as normalization controls. (D) WT or TLR4−/− mouse DCs were treated RPS3 (1 μg/mL) and then pulsed with the E7 tumor antigen peptide. Following this the DCs injected were injected into the footpads of mice. The number of tumor-specific CD8^+^IFN-γ^+^ T cells was then determined by flow cytometry. (E) After vaccination with the prepared WT or TLR4−/− DCs, TC-1 cells were subcutaneously injected and tumor mass and mouse survival were analyzed. All the experiments were performed three times. IBM SPSS Statistics Base 22.0 was used as statistical method to analyze differences between experimental groups. (*; *p* < 0.05, **; *p* < 0.01 and ***; *p* < 0.001)

(A) Schematic showing the two fragments of the RPS3 protein (1–95 and 91–243). (B) The two His-tagged fragments of the RPS3 protein was assessed for purity and size by western blot. (C) The different forms of the RPS3 protein (WT, 1–95, and 91–243; 1 μg/mL), GFP (5 μg/mL), or LPS (100 ng/mL) were used to treated 293/hTLR4A-MD2-CD14 cells, after which luciferase activity was measured. (D, E) The different forms of the RPS3 protein (WT, 1–95, and 91–243) (1 μg/mL), GFP (5 μg/mL), or LPS (100 ng/mL) were used to treat mouse BMDC and the levels of secreted cytokines (TNF-α, IL-1β, IL-6, IL-10, IL-12p70 and IFN-β), as well as the expression of co-stimulatory molecules (CD40, CD80, CD86, and MHC-I) were measured. All the experiments were performed three times. IBM SPSS Statistics Base 22.0 was used as statistical method to analyze differences between experimental groups. (N.S.: not significant, *; *p* < 0.05, **; *p* < 0.01 and ***; *p* < 0.001)

## Results

### Release of RPS3 from tumor cells and the binding of recombinant RPS3 from *E. coli* to recombinant TLR4

**Fig. 1:**
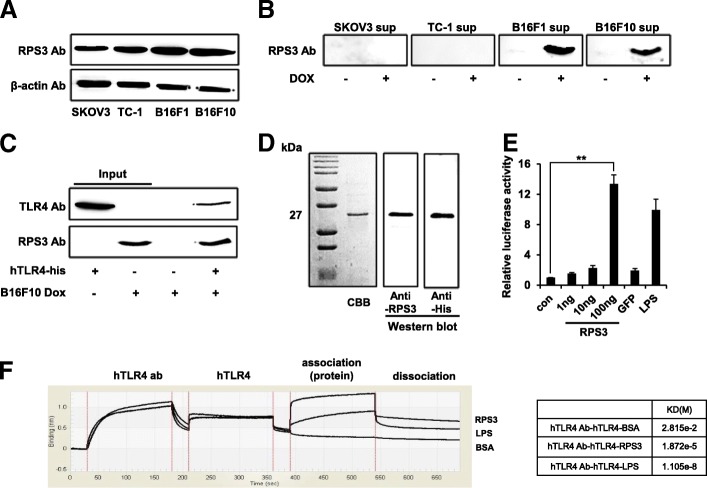
The association of TLR4 with released or recombinant RPS3

To identify protein candidates in human cancer cells that can associate with TLR4, we screened human cancer cells using a luciferase assay and three cancer cell lines were selected in which NF-kB activity could be observed (Additional File 2: Figure S1). Following this, lysates from three cancer cells were used in pulled-down experiments with recombinant TLR4 (Additional File 2: Figure S2). Among the various ribosomal protein families that were found to bind to TLR4, ribosomal protein S3 (RPS3) was selected for use in our experiments because it had the greatest effects when used to treat BMDCs. An initial experiment revealed that RPS3 is expressed in various cancer cells (Fig. 1A). Furthermore, RPS3 was released from not only B16F1 and B16F10 tumor cells (Fig. 1B) but also normal cells like BMDCs (Additional File 2: Figure S3) when they were treated with doxorubicin and the released RPS3 could bind to TLR4 (Fig. 1C). SKOV3 supernatant does not seem to release RPS3 due to merely effects of doxorubicin occurring cell death in SKOV3 compared to other tumor cells (Fig. 1B). Recombinant RPS3 was then purified (Fig. 1D) and the interaction between RPS3 and TLR4 was confirmed by the increased luciferase activity seen after TLR4-MD2 cells were treated with RPS3 (Fig. 1E). Using a blitz assay, the Kd (M) value for the RPS3 and TLR4 interaction was lower (i.e. of higher affinity) than the BSA and TLR4 interaction (Fig. 1F). It is concluded that RPS3 is released from tumor cells when treated with an anticancer drug and that RPS3 can associate with TLR4.

### RPS3 induces maturation and activation of mouse BMDCs, THP-1 cells, and human DCs

**Fig. 2:**
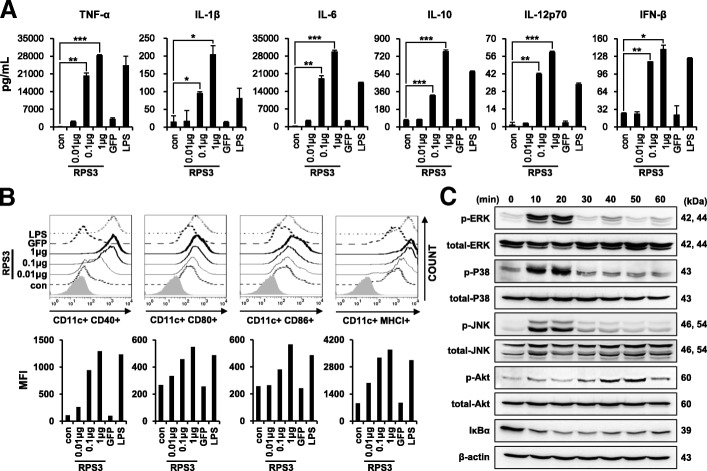
Maturation and activation of mouse dendritic cells by recombinant RPS3

To confirm the effects of RPS3 which binds to TLR4, we used recombinant RPS3 from E.coli in all experiments due to productivity and convenience. Firstly, we determined the effects of RPS3 on mouse BMDCs by characterizing the expression of pro-inflammatory cytokines and IFN-β. The levels of TNF-α, IL-1β, IL-6, IL-10, IL-12p70, and IFN-β were all significantly increased in a dose-dependent manner in DCs treated with RPS3 compared to untreated DCs (Fig. 2A). In addition, the expression levels of co-stimulatory molecules CD40, CD80, CD86, and MHC class I were also increased in DCs treated with RPS3 compared to untreated DCs (Fig. 2B). To evaluate the activation of signaling pathways, mouse BMDCs were treated with RPS3 in a time course over an hour. The levels of p-ERK, p-P38, and p-JNK and p-AKT were all found to be elevated (Fig. 2C). To assess NF-κB activation, we measured the degradation of the inhibitory IκB-α protein (Fig. 2C). The activation of DCS by RPS3 was carried out using protein purified from *E. coli* in such a way that endotoxins were removed from the preparation. As a negative control, GFP prepared in an identical manner was used; LPS was used as a positive control since it is a known TLR4 ligand. We assessed LPS contamination of the RPS3 protein preparation used to treat DCs and showed that it was not as a result of endotoxin contamination from *E. coli*. Treatment with polymyxin B (PMB) decreased the effects of LPS in DCs, whereas the effects of RPS3 in DCs were not affected but instead were decreased by proteinase K treatment (Additional File 2: Figure S4). These results indicate that RPS3 can induce the secretion of pro-inflammatory cytokines, increase the expression of co-stimulatory molecules, and activate signaling pathways in mouse BMDCs.

**Fig. 3:**
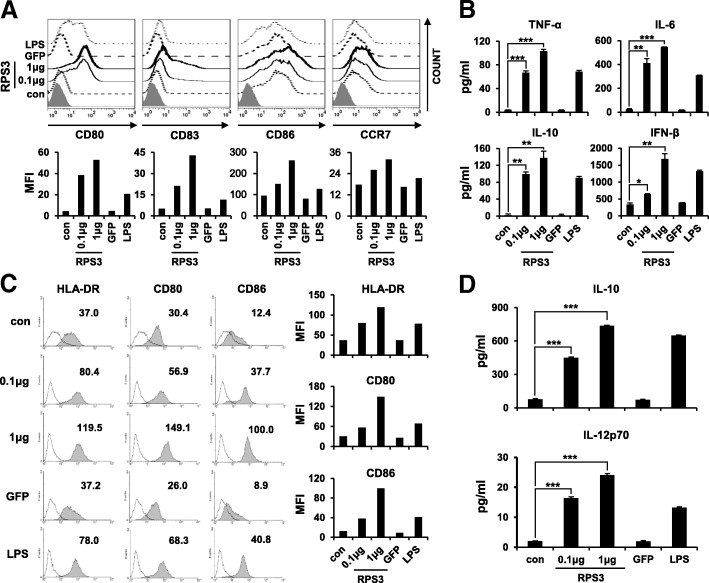
Maturation and activation of human monocytic THP-1 cells and human dendritic cells by RPS3 protein

Next, we assessed the effects of RPS3 on the human monocytic cell line THP-1, as well as human DCs. As shown in Fig. 3A, the expression levels of CD80, CD83, CD86, and the migration factor CCR7 were all increased in a dose-dependent manner in THP-1 cells treated with RPS3 compared to untreated THP-1 cells. Moreover, the secretion levels of the pro-inflammatory cytokines TNF-α, IL-6, IL-10, and IFN-β were increased in THP-1 cells treated with RPS3 compared to untreated THP-1 cells (Fig. 3B). Likewise, the expression of the co-stimulatory molecules MHC class II, CD80, and CD86, as well as the of the pro-inflammatory cytokines IL-10 and IL-12, were all increased in RPS3-treated human DCs (Fig. 3C and D). Interestingly, the expression levels of co-stimulatory molecules and the secretion levels of pro-inflammatory cytokines were higher in RPS3-activated THP-1 cells and human DCs than in LPS-activated THP-1 cells or human DCs. These results suggest that RPS3 can induce the activation and maturation of human monocytes and human DCs.

### RPS3-activated BMDC vaccine produces antigen-specific CD8^+^ T cells in vivo and leads to tumor prevention, effective tumor treatment, and the generation of memory T cells

**Fig. 4:**
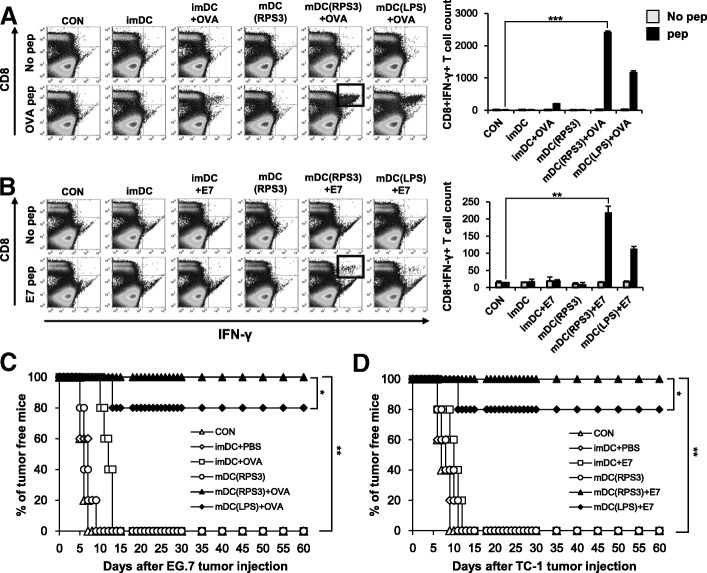
In vivo study showing tumor specific CD8^+^IFN-γ^+^ T cell induction and tumor prevention effects with a DC-based cancer vaccine using the RPS3 protein as adjuvant

We next assessed the effects of mature DCs activated by RPS3 as a DC vaccine to assess whether RPS3-activated DCs can generate antigen-specific CD8^+^ T cells. Mouse BMDCs (2 × 10^6) were treated with RPS3 or LPS after which the DCs were pulsed with a tumor specific peptide and then injected into the footpad of mice once a week for two weeks. Following this, the number of IFN-γ secretory CD8^+^ T cells was measured. As shown in Fig. 4A and B, tumor specific CD8^+^IFN-γ^+^ T cells were significantly generated in a mouse vaccinated with RPS3-activated DCs pulsed with either OVA or E7 antigenic peptides, as compared to mice vaccinated with immature DCs or non-peptide pulsed mature DCs (OVA and E7 are well known antigens expressed in EG.7 and TC-1 cells respectively). Next, we assessed if these RPS3-activated DC vaccines could prevent tumorigenesis. The RPS3-activated DCs were pulsed with the tumor specific antigens OVA or E7 and were then injected into the footpads of mice. After the last vaccination, EG.7 or TC-1 cells were then injected subcutaneously into the mice. As shown in Fig. 4C and D, all the mice vaccinated with RPS3-activated DCs pulsed with the OVA or E7 peptides remained tumor-free for 60 days. In contrast, tumor formation occurred within 15 days in mice vaccinated with immature DCs pulsed with peptide or mature DCs that were not pulsed with peptide or mature DCs pulsed with non-specific peptide (Additional File 2: Figure S5 A). Interestingly, the RPS3-activated DCs pulsed with OVA or E7 peptide vaccine had a slightly greater effect than the LPS-activated DCs pulsed with OVA or E7 peptide vaccine.

**Fig. 5:**
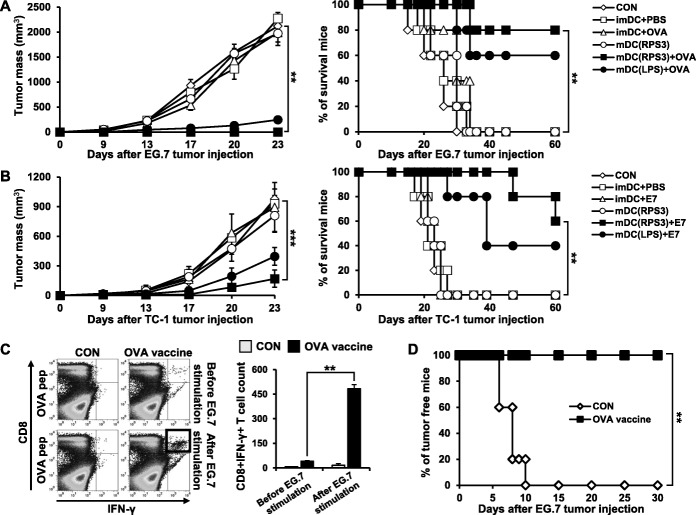
In vivo study showing the tumor treatment effect and the generation of memory T cells by the DC-based cancer vaccine using RPS3 as adjuvant

Next, we evaluated the effect of the RPS3-treated DC vaccine on tumor treatment. EG.7 or TC-1 cells were injected subcutaneously into mice, and after establishment of a tumor mass on day 5, RPS3-treated DCs pulsed with OVA or E7 were used to vaccinate the mice using a footpad inoculation once a week for two weeks. As shown in Fig. 5A and B, a suppression of tumor growth and the long-term survival was seen in mice injected with RPS3-treated DCs pulsed with OVA or E7 compared to mice injected with untreated DCs or RPS3-treated DCs not pulsed with peptide. More importantly, the tumor treatment effects in mice vaccinated with the RPS3-treated DCs pulsed with OVA or E7 were greater than in mice vaccinated with LPS-treated DCs pulsed with OVA or E7 (Fig. 5A and B). To understand the function of memory T cells, the tumor cells were injected seven weeks after DC vaccination. The number of CD8^+^IFN-γ^+^ T cells was dramatically increased in mice which had been vaccinated with BMDCs treated with RPS3 and pulsed with the OVA peptide (Fig. 5C) rather than non-specific peptide E7 (Additional File 2: Figure S5 B). Long-term prevention effects were also shown as vaccinated mice remained tumor free. However, tumors continued to grow in unvaccinated mice (Fig. 5D). Therefore, a vaccine comprised of DCs treated with RPS3 and pulsed with a tumor specific peptide has a significant effect in producing memory T cells. Furthermore, we clarified which cytotoxic immune cell make the most significant contribution to the effects of the RPS3-based DC vaccine on tumor prevention using depletion with antibodies against CD4, CD8, and NK (Additional File 2: Figure S6). As a result, the survival of mice vaccinated with RPS3-activated DCs decreased when the mice were also injected with a CD8-depleting antibody, indicating that CD8^+^ T cells function to prevent tumor formation and have an antitumor effect.

### RPS3-mediated activation and maturation of DCs and its adjuvant effect in DC vaccine are dependent on TLR4

**Fig. 6:**
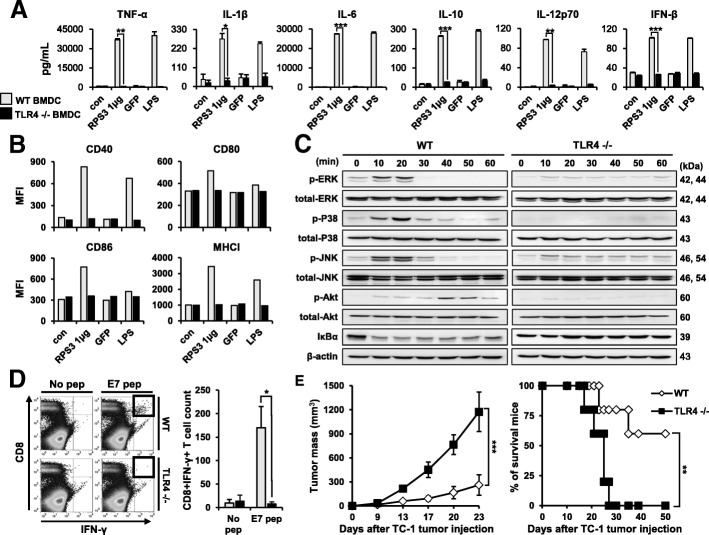
Comparison of RPS3 protein activity in WT and TLR4−/− mouse BMDCs

Next, we examined the TLR4-dependency for DC activation and maturation, and its adjuvant effects in the DC vaccine. Mouse BMDCs from either wild type or TLR4−/− mice were treated with RPS3 or LPS. After 16 h of treatment, the levels of the secreted pro-inflammatory cytokines TNF-α, IL-1β, IL-6, IL-10, IL-12, and IFN-β were evaluated by ELISA. As shown in Fig. 6A, DCs treated with RPS3 or LPS showed the expected increases in cytokine levels and co-stimulatory molecules (CD40, CD80, CD86, and MHC class I) compared to untreated cells. These increases were not observed in TLR4−/− DCs. And the same effects were shown with a TLR4 blocking antibody as was shown with TLR deficient mice. The expression of co-stimulatory molecule CD40 and the secretion of pro-inflammatory cytokines TNF- α and IL-6 were decreased after pre-treated by TLR4 blocking antibody (Additional File 2: Figure S7). In addition, the levels of p-ERK, p-P38, p-JNK, and p-AKT were increased and the levels of IкB-α were decreased in wild type DCs after incubating with RPS3 in a time course over an hour, there was no effect on signaling pathways in TLR4 −/− DCs. Finally, the effect of the DC-based cancer vaccine was assessed using wild type and TLR4−/− BMDCs to see whether its adjuvant effects are dependent on TLR4. The number of tumor specific CD8^+^IFN-γ^+^ T cells was greatly increased when mice were vaccinated with RPS3-activated wild type DCs pulsed with E7, but not with RSP3 treated TLR4−/− DCs pulsed with E7. To examine the therapeutic anti-tumor effects of these two vaccines, mice were first injected with TC-1 tumor cells to establish tumor mass. After the tumor mass was established, the mice were vaccinated with either RPS3 treated wild type or TLR4−/− DCs pulsed with E7 peptide once a week for two weeks. As shown in Fig. 6E, the tumor treatment effect was absent in mice injected with the RPS3-treated DCs lacking TLR4 and pulsed with E7. These observations show that TLR4 is necessary for DC activation and maturation by RPS3 and for its ability to function as an adjuvant in the DC vaccine.

### Two fragments of RPS3 (1–95 and 91–243) have different effects on BMDCs

**Fig. 7:**
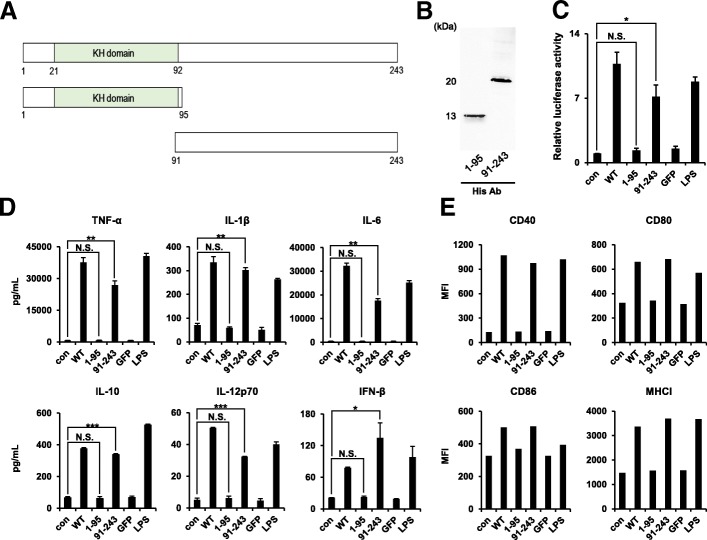
Identification the RPS3 protein domain associated with TLR4 signaling

After characterizing the ability of RPS3 to induce maturation and activation of DCs, we sought to perform further functional studies to assess which portion of RSP3 is important for the adjuvant effects. The KH domain, (amino acids from 22 to 92 in RPS3 (1–243)), is a well-known DNA & RNA binding domain. On the basis of the presence of this KH domain, two fragments of RPS3, a fragment (1–95) and another fragment (91–243), were cloned and generated (Fig. 7A). Each His-tagged fragment (1–95, 91–243) was purified from *E. coli* and confirmed by western blot (Fig. 7B). TLR4-MD2 overexpressing HEK293 cells were then treated with RPS3, one fragment (1–95), or the other fragment (91–243). As shown in Fig. 7C, luciferase activity was increased by RPS3 and the (91–243) fragment, whereas there was no effect of the (1–95) fragment. The levels of secreted pro-inflammatory cytokines TNF-α, IL-1β, IL-6, IL-10, IL-12 and IFN-β were significantly increased in DCs treated with RPS3 or the (91–243) RPS3 fragment, but not following treatment with the (1–95) fragment (Fig. 7D). The expression levels of the co-stimulatory molecules CD40, CD80, CD86, and MHC class I were also increased in DCs treated with the (91–243) RPS3 fragment (Fig. 7E). Therefore, these results suggest that the effect of RPS3 as an adjuvant for DC stimulation is mediated mainly by the 91–243 fragment (which does not include the KH domain). This small protein fragment protein might therefore be useful in immunotherapy.

## Discussion

In this study, we sought to find new ligands for TLR4. First, a number of intracellular proteins extracted from different human cancer cells showed a significant increase in NF-кB activity when used to treat HEK293 cells expressing TLR4-MD2. Using these human cancer cells, numerous ribosomal family proteins were found in pull-down assays conducted with recombinant TLR4. After purification and treatment of mouse BMDCs to assess the TLR4 ligand candidates, ribosomal protein S3 (RPS3) stood out as the strongest inducer of maturation and activation of DCs. Accordingly, we used RPS3 as an adjuvant in a DC-based vaccine to generate CD8^+^ T cells and to prevent and treat tumors. Notably, RPS3 had large effects on DCs, even at very low concentrations (0.1 μg/mL) compared to HMGB1, a DNA binding protein, which requires 50~100 μg/mL to induce maturation and activation of DCs [31]. Since HMGB1 is well-known TLR4 binder, HMGB1 was used directly to compare with the effects of RPS3 by activating BMDCs (Additional File 2: Figure S8).

RPS3 exists intracellularly in both mouse and human cancer cells, but it is secreted after its N-linked glycosylation [28]. Based on previous studies, the intracellular protein HMGB1 is known to be secreted from tumor cells following chemotherapy or radiotherapy, and it is also known that HMGB1 can bind to TLR4 on DCs and activate downstream signaling pathways [32]. In this study, we also demonstrated that RPS3 is released from tumor cells following treatment with an anti-cancer drug, and that this released RPS3 can affect the surrounding DCs expressing TLR4 in the tumor microenvironment. To confirm that RPS3 binds to TLR4 and activates DCs, recombinant human RPS3 was purified and used to treat human and mouse DCs. As a result, human-derived RPS3 had an excellent effect on both species of DCs even though a human-derived protein can sometimes have different effects on mouse DCs. Human RPS3 is 99% identical to mouse RSP3 and so the effects of RPS3 in human and mouse dendritic cells are likely to be the same [33]. Furthermore, since RPS3 is expressed and released by tumor cells, DC would be activated by tumor lysate which contains RPS3. However, tumor lysates contain varied DAMPs or inflammatory factors even except RPS3 that can activate DCs (Additional File 2: Figure S9).

Next, we demonstrated that the number of tumor specific CD8^+^IFN-γ^+^ T cells is increased and that both tumor prevention and tumor treatment effects could be achieved using a tumor-specific antigen pulsed DC-based vaccine with RPS3 as an adjuvant. Even though the DC-based vaccine had a large effect on tumors, larger size or terminal cancers might require alternative treatments. We expect better effects when immune checkpoint inhibitors are used together with a DC-based vaccine. Immune checkpoint inhibitors such as Ipilimumab, a CTLA-4 inhibitor for the treatment of melanoma, is already approved by the FDA. In addition, several other immune checkpoint inhibitors, such as PD-1 and PD-L1, are undergoing clinical trials for the treatment of several cancers, including melanoma, lung, and breast cancer [34]. Currently, we are investigating the use of this DC-based vaccine together with immune checkpoint inhibitors to examine if they have synergistic or greater effects. Furthermore, RPS3 is confirmed to be safe when using as an adjuvant in tumor-specific antigen DC-based vaccines via TLR4 since RPS3 is nucleoprotein that could induce humoral immunity, producing autoantibodies against itself (Additional File 2: Figure S10).

We also examined the effects of the RPS3 protein on DCs and the dependency on TLR4 using TLR4−/− and wild type mice. We suspected that if DNA binds to the KH domain of RPS3 this could induce responses through TLR3, TLR7, or TLR9. Therefore, two fragments (1–95 and 91–243) were constructed based on the location of the KH domain in RPS3 to understand whether the KH domain of RPS3 directly participates in DC maturation and activation. When the two RPS3 fragments (1–95 containing the KH domain and 91–243 lacking the KH domain) were used to treat HEK293-MD2-TLR4 cells or BMDCs, only by the (91–243) fragment increased luciferase activity and stimulated BMDCs. Therefore, based on the TLR4 dependency, and the fact that the (91–243) fragment, which lacks the DNA binding KH domain, is active we can conclude that DNA binding is not related to the effects on DCs.

Based on the findings of this study, we conclude that RPS3 from human cancer cells has a very good effect on the immune system. RPS3 induces maturation and activation of dendritic cells, acting as a new ligand for TLR4 in the innate immune system, and significantly increases CD8^+^ T cell production in the presence of a tumor-specific antigen in the adaptive immune system. Furthermore, since RPS3 is released when tumors are treated by chemotherapy, it is necessary for us to develop RPS3 neutralizing antibody to block TLR4/RPS3 interaction in vivo to demonstrate a more specific mechanism. In addition, RPS3 is a human-derived protein that is safe for in vivo use, unlike LPS, a bacterial endotoxin, and has been shown to be highly effective in very small doses when compared to other adjuvants that can cause maturation and activation of DCs. Thus, RPS3 is a novel and potential adjuvant for DC-based cancer vaccines.

In our previous studies, not only RPS3, but also several ribosomal proteins that bind to TLR4 were screened from human tumor cells. A study on the effects of these ribosomal proteins to induce maturation and activation of DCs has revealed previously unknown functions of these other ribosomal proteins on DCs. Therefore, we are currently assessing the potential of these other ribosomal proteins for use as novel adjuvants for the development of DC-based vaccines.

## Additional files

Additional file 1: Supplementary Materials and methods. (DOCX 20 kb)

Additional file 2: **Figure S1.** NF-κB activity was measured using cancer cell lysates to assess TLR4-expressing tumor cells. **Figure S2.** To identify the TLR4 binding proteins, two groups were divided and assessed through pull-down assay. **Figure S3** Mouse BMDCs were treated with doxorubicin (0, 0.1, 1, 10 μg/mL). **Figure S4.** For endotoxin contamination test, recombinant RPS3 (1 μg) and LPS (100 ng) were incubated with proteinase K (100 μg/ml) or with polymyxin B (10 μg/ml). **Figure S5** Mouse BMDCs were separated into RPS3 treated mature DCs loaded with OVA peptide or E7 peptide. **Figure S6.** To confirm the dominancy of T cells in adaptive immune responses to clear tumors, T cell or NK cell depletion antibodies were injected into mice before the injection of cancer cells. **Figure S7** We examined the TLR4-dependency for DC activation and maturation by using TLR4 blocking antibody. **Figure S8** The purification of recombinant HMGB1 protein was confirmed by CBB staining and by western blot (A). **Figure S9** HEK293 cells were transfected by shRNA (GFP) as a negative control or shRNA (RPS3). **Figure S10** Mice serum with vaccination or not were used to confirm that RPS3 does not induce humoral immunity, producing autoantibodies against itself. (DOCX 1509 kb)
