# Supplementary material for: A novel TLR4 binding protein, 40S ribosomal protein S3, has potential utility as an adjuvant in a dendritic cell-based vaccine
Source: J Immunother Cancer. 2019 Feb 28;7:60. doi: 10.1186/s40425-019-0539-7 (PMC6394096; doi:10.1186/s40425-019-0539-7)
Supplement: Supplementary file 1 — Supplementary Materials and methods. (DOCX 20 kb) [file 40425_2019_539_MOESM1_ESM.docx]

**Methods**

**Cells**

Human DCs were differentiated after the isolation of monocytes from peripheral blood mononuclear cells (PBMCs) isolated from healthy donors according to the approved protocol from the Institutional Research Board of Chonnam National University Hwasun Hospital. Cells were isolated using a CD14^+^ magnetic activating cell sorting (MACs) system (Milenyi Biotec Inc., Auburn, CA, USA), after which 2 × 10^5^ cells/well were incubated in 6-well plates (BD Falcon, San Jose, CA, USA) in RPMI-1640 medium (Gibco-BRL; Grand Island, NY, USA) supplemented with 10% fetal bovine serum (Hyclone; Logan UT, USA), 1% penicillin streptomycin, 50 ng/mL recombinant human granulocyte macrophage colony-stimulating factor (LG Biochemicals, Daejon, South Korea), and 20 ng/mL recombinant human IL-4 (R&D Systems; Minneapolis, MN, USA). Culture medium was changed every two days and the cells were differentiated for six days.

**Purification of recombinant RPS3 protein**

The pET28b-RPS3 plasmid was transformed into *Escherichia coli* Rosetta competent cells (Novagen, MA, USA). *E. coli* Rosetta bacterial cells expressing the RPS3 protein were incubated at 37°C for 5 h, after which 1 mM IPTG (isopropyl β-D-1-thiogalactopyranoside, BioBasic, Markham ON, Canada) was added and the culture continued at 25°C for 12 h. The *E. coli* were centrifuged at 6000 rpm for 10 min and the resulting pellet was lysed with SoluLyse pH 7.4 (Genlantis, CA, USA), 5U/mL deoxynuclease Ⅰ (BioBasic, Markham ON, Canada), 50 μg/mL lysozyme (BioBasic, Markham ON, Canada), and 5 mM DTT (BioBasic, Markham ON, Canada) at room temperature for 2 h. The lysed *E. coli* cells were centrifuged at 12000 rpm for 15 min, the cell pellets collected and lysed on ice for 1 h in 6 M urea buffer, and the lysate centrifuged at 13000 rpm for 15 min. The supernatant was then filtered using a 0.45-μm filter and incubated with Ni-NTA agarose beads (Qiagen, Hilden, Germany) at 4°C overnight. Following this, RPS3 was eluted using PBS buffer containing imidazole and 6 M urea. The purified protein was then dialyzed to remove any excess imidazole and urea. Contaminating endotoxin was then removed using Triton X-114 Surfact-Amps solution (Thermo Scientific, Massachusetts, USA) until the amount of endotoxin was less than 0.1 EU/mg, as assessed using an LAL assay kit (Lonza, Basel, Swiss). Successful purification of the RPS3 protein was confirmed by staining of Coomassie Brilliant Blue SDS-PAGE gels and western blot analysis with an anti-RPS3 antibody (Novus, Colorado, USA) and an anti-His antibody (MBL, MA, USA).
 The purification of GFP was performed by transforming the pET28a-GFP plasmid into *E. coli* BL21(DE3) competent cells (NEB, MA, USA). *E. coli* BL21(DE3). GFP expression was induced at 37°C by the addition of 1mM IPTG and culturing for 5 h. The subsequent purification and analytical steps were performed using the same conditions used for the RPS3 protein. And the recombinant RPS3 from E.coli was purified at the same time and being stored at -70°C which was used in all experiments. The recombinant RPS3 protein was confirmed without LPS contamination through LAL test.

**Western blotting**

Mouse BMDCs were treated with RPS3 (1 μg/mL) over a time course (0, 10, 20, 30, 40, 50, and 60 min) and the cells harvested. Cells were lysed using RIPA buffer (0.5% NP-40, 1 mM EDTA, 50 mM Tris-Cl pH 8.0, 120 mM NaCl, protease inhibitor cocktail, 100 mM PMSF, 0.5 M NaF) on ice for 1 h.

**Pull-down assay**

For TLR4-treated cancer cells, 50 μL of Ni-NTA beads (Qiagen, Hilden, Germany), and 10 μg of hTLR4-His (Sino, New Jersey, USA), were added to 50 μg of cancer cell lysate. For non-TLR4-treated cancer cells, as a negative control, 50 μL of Ni-NTA beads were mixed with 50 μg of cancer cell lysate. All binding experiments were performed in 1 × PBS buffer (pH 6.0). The resin was then washed five times and then boiled at 100°C in 1 × SDS loading buffer. After electrophoresis on 12% SDS-PAGE gels, the proteins were stained using a silver stain and the TLR4 binding protein were identified using a MALDI-TOF MS analysis system and the MASCOT search program (Korea Basic Science Institute, Daejeon, South Korea).

***In vitro* endotoxin contamination test**

Recombinant RPS3 (1 μg) and LPS (100 ng) were incubated with proteinase K (100 μg/mL) (NEB, MA, USA) at 37°C for 16 h or with polymyxin B (10 μg/ml) (Sigma, Missouri, USA) at 4°C for 10 min. Proteinase K activity was inhibited by heating at 95°C for 10 min. The pre-treated RPS3 and LPS were then used to treat mouse dendritic cells. An analysis of DC maturation and activation was performed using flow cytometry and the cell surface markers CD40 and MHC-I, and using ELISA assays for the cytokines TNF-α and IL-6.

***In vivo* experiments**

For the T cell or NK cell depletion experiments, anti-CD4 (MAb GK1.5), anti-CD8 (MAb 2.43), or anti-NK1.1 (MAb PK136) depletion antibodies were injected into the peritoneum of mice every two days starting from three days before the injection of cancer cells. After tumor cell injection, the mice were observed until the tumor diameter was over 3 mm.
