# Supplementary material for: A novel TLR4 binding protein, 40S ribosomal protein S3, has potential utility as an adjuvant in a dendritic cell-based vaccine
Source: J Immunother Cancer. 2019 Feb 28;7:60. doi: 10.1186/s40425-019-0539-7 (PMC6394096; doi:10.1186/s40425-019-0539-7)
Supplement: Supplementary file 2 — Figure S1. NF-κB activity was measured using cancer cell lysates to assess TLR4-expressing tumor cells. Figure S2. To identify the TLR4 binding proteins, two groups were divided and assessed through pull-down assay. Figure S3 Mouse BMDCs were treated with doxorubicin (0, 0.1, 1, 10 μg/mL). Figure S4. For endotoxin contamination test, recombinant RPS3 (1 μg) and LPS (100 ng) were incubated with proteinase K (100 μg/ml) or with polymyxin B (10 μg/ml). Figure S5 Mouse BMDCs were separated into RPS3 treated mature DCs loaded with OVA peptide or E7 peptide. Figure S6. To confirm the dominancy of T cells in adaptive immune responses to clear tumors, T cell or NK cell depletion antibodies were injected into mice before the injection of cancer cells. Figure S7 We examined the TLR4-dependency for DC activation and maturation by using TLR4 blocking antibody. Figure S8 The purification of recombinant HMGB1 protein was confirmed by CBB staining and by western blot (A). Figure S9 HEK293 cells were transfected by shRNA (GFP) as a negative control or shRNA (RPS3). Figure S10 Mice serum with vaccination or not were used to confirm that RPS3 does not induce humoral immunity, producing autoantibodies against itself. (DOCX 1509 kb) [file 40425_2019_539_MOESM2_ESM.docx]

**Figure S1**


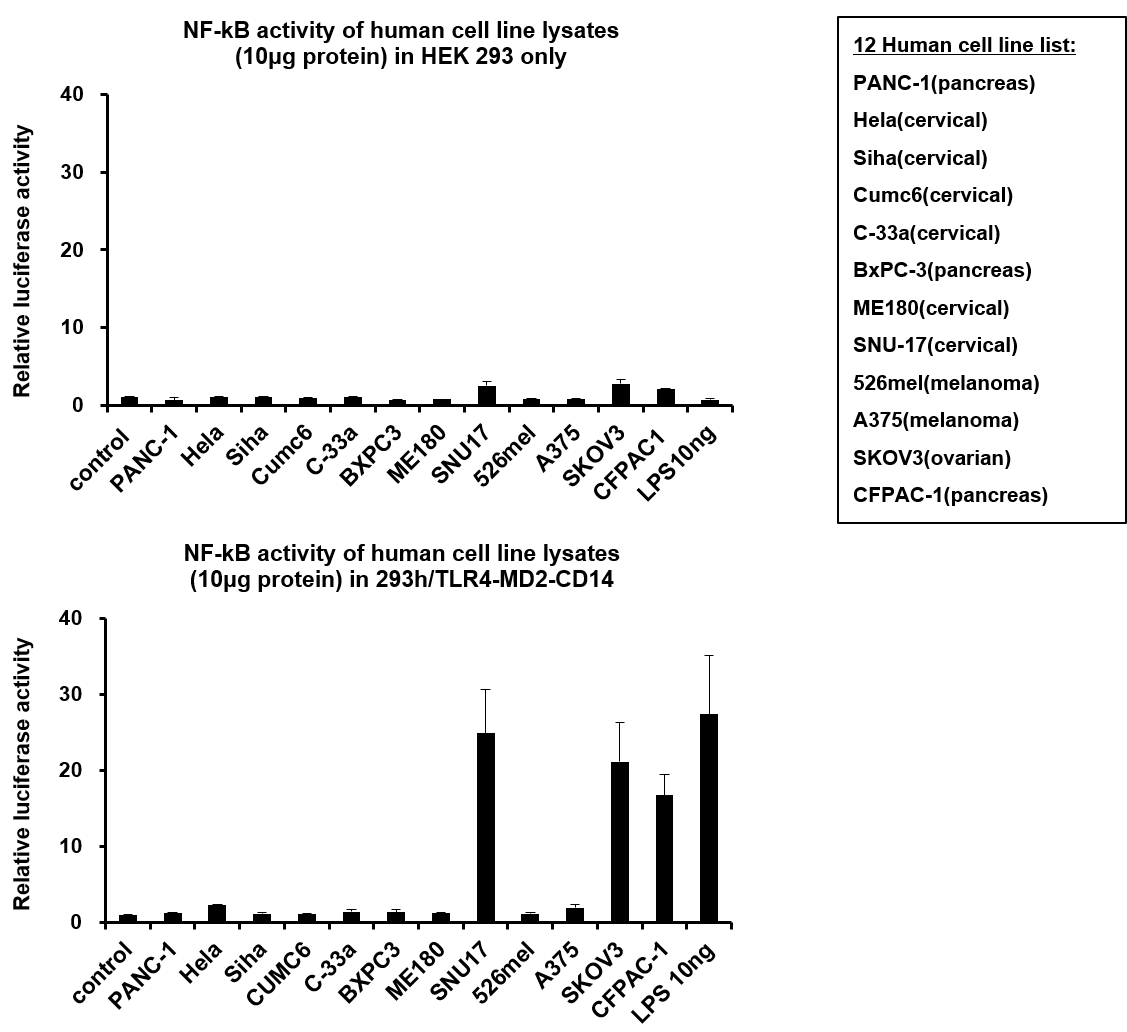


NF-κB activity was measured using cancer cell lysates to assess TLR4-expressing tumor cells. The lysed human cells were quantified and treated on HEK 293 expressing TLR4-MD2-CD14 or not. Among 12 human cell lines, the relative luciferase activity was significantly increased in SNU17, SKOV3, and CFPAC-1 in a TLR4 dependent manner.

**Figure S2**


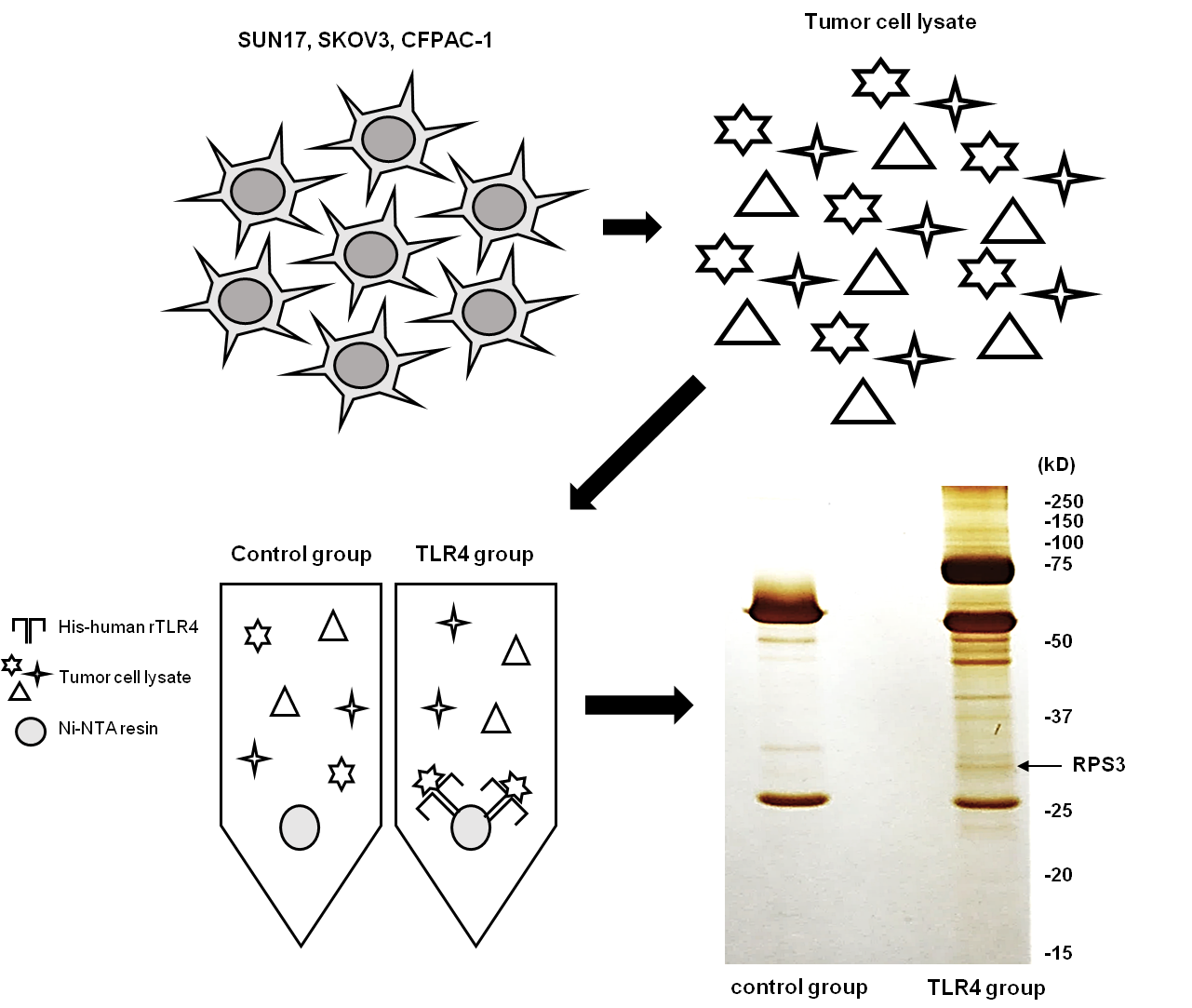


To identify the TLR4 binding proteins, two groups were divided and assessed through pull-down assay. For TLR4-treated cancer cells, 50 μL of Ni-NTA beads and 10 μg of hTLR4-His were added to 50 μg of cancer cell lysate. For a negative control, 50 μL of Ni-NTA beads were added to 50 μg of cancer cell lysate in which no hTLR4-His was mixed. The resin was washed and boiled in 10x SDS loading buffer. After electrophoresis, the proteins were stained using a silver stain and the TLR4 binding proteins were identified.

**Figure S3**

**
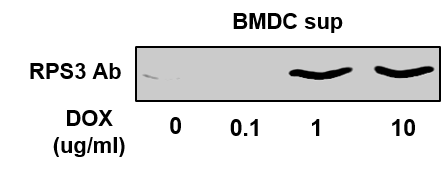
**

Mouse BMDCs were treated with doxorubicin (0, 0.1, 1, 10 μg/mL). After 2 h, the media was changed to Opti-MEM media and the cells were incubated overnight. Release of RPS3 into the culture supernatant was assessed by western blot.

**Figure S4**


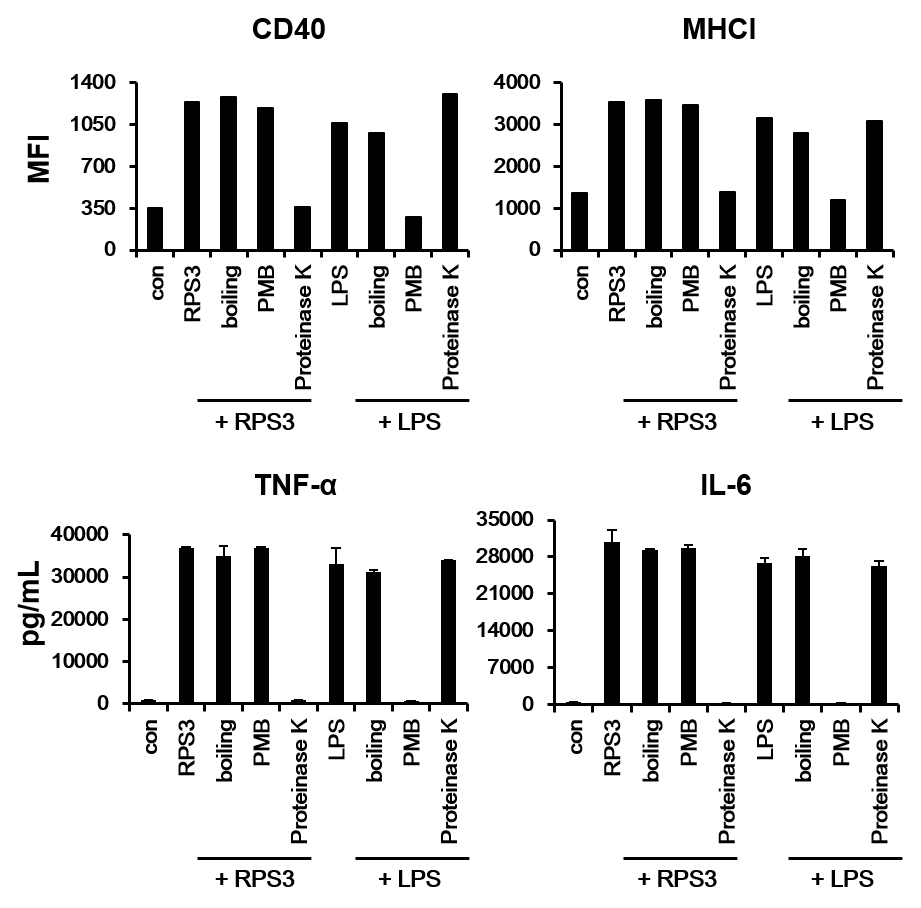


For endotoxin contamination test, recombinant RPS3 (1 μg) and LPS (100 ng) were incubated with proteinase K (100 μg/ml) or with polymyxin B (10 μg/ml). After treatment, DC maturation and activation was analyzed by flow cytometry and ELISA.

**Figure S5**

**
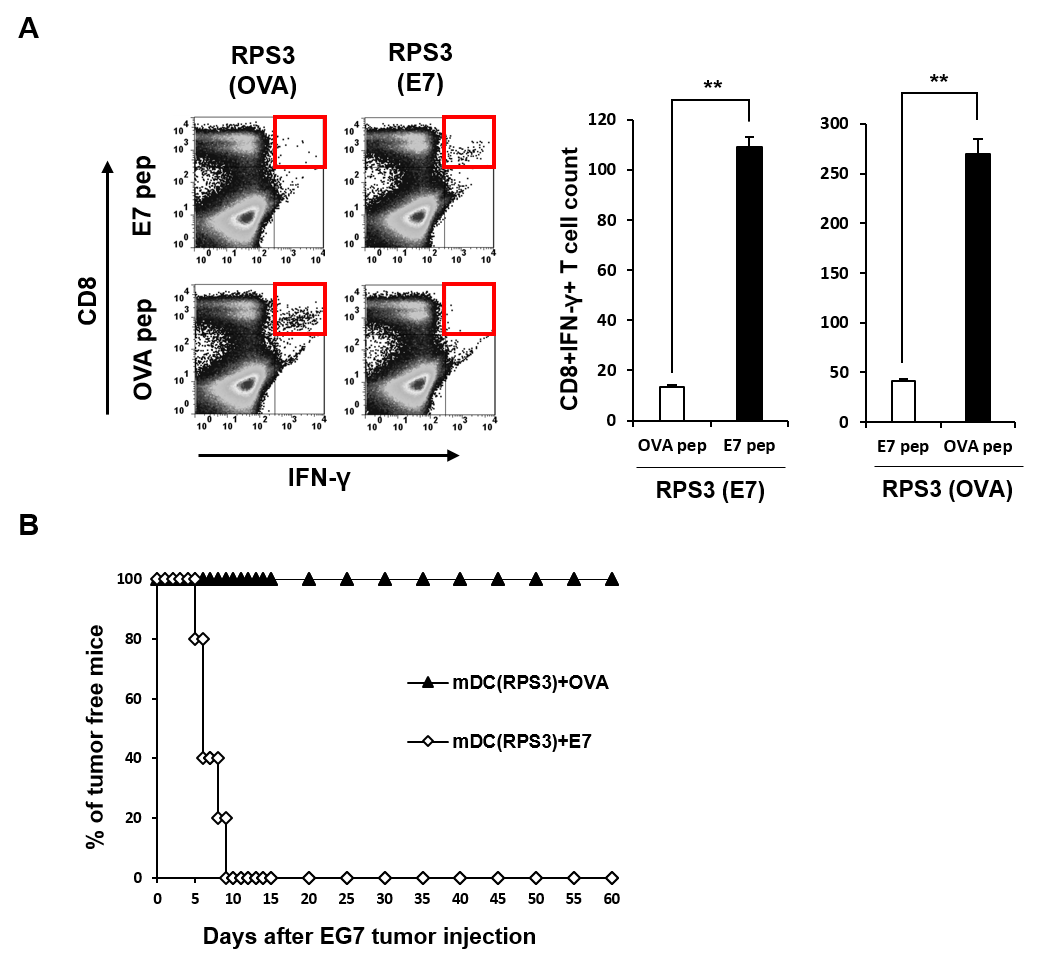
**

Mouse BMDCs were separated into RPS3 treated mature DCs loaded with OVA peptide or E7 peptide. DCs were treated with RPS3 (1 μg/mL) and then pulsed with OVA or E7 tumor specific antigen peptides. The treated DCs were then injected into the footpads of mice. The number of tumor specific CD8^+^IFN-γ^+^ T cells produced in the mouse spleen was counted by flow cytometry (A). Mouse BMDCs were separated into the following groups: vaccination with OVA peptide or E7 peptide following EG7 tumor injection. Seven weeks after the last BMDC injection, EG.7 tumor cells were injected subcutaneously into mice and tumor formation was observed (B).

(**; p < 0.01 and ***; p < 0.001)

**Figure S6**


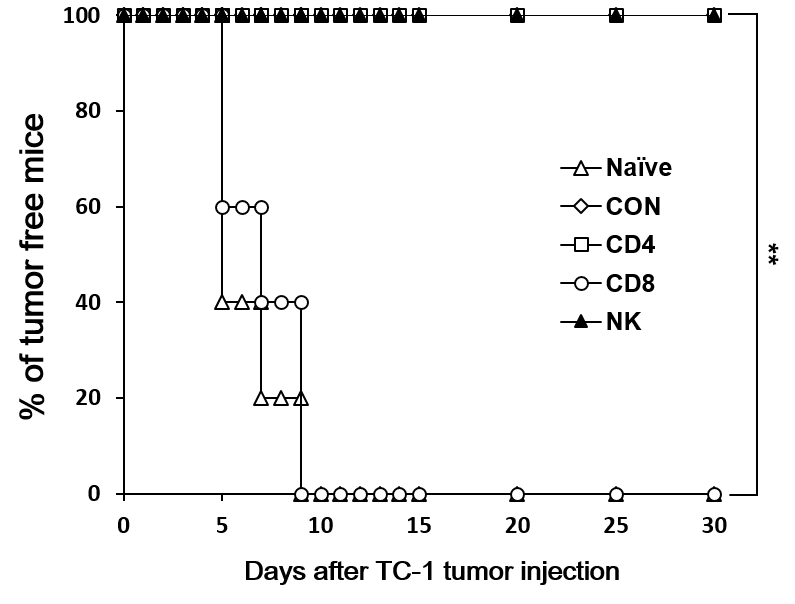


To confirm the dominancy of T cells in adaptive immune responses to clear tumors, T cell or NK cell depletion antibodies were injected into mice before the injection of cancer cells. The mice were observed until the tumor diameter was over 3 mm.

**Figure S7**

**
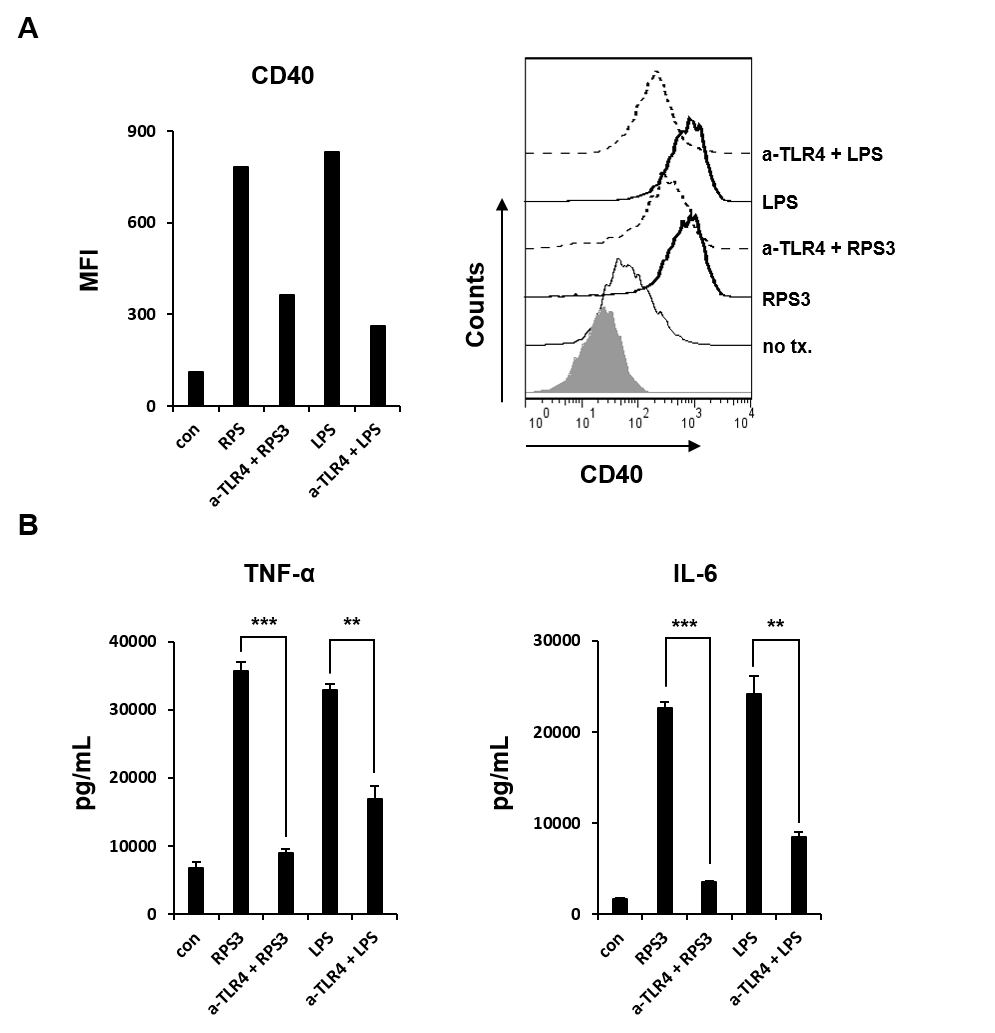
**

We examined the TLR4-dependency for DC activation and maturation by using TLR4 blocking antibody. Firstly, the expression levels of co-stimulatory molecule CD40 was increased in DCs treated with RPS3 or LPS compared to untreated DCs. And these increases were not observed in DCs treated with RPS3 or LPS after TLR4 blocking antibody (50ug/ml) (A). Next, the levels of the secreted pro-inflammatory cytokines TNF- α and IL-6 were evaluated by ELISA. As expected, DCs treated with RPS3 or LPS showed the increased in cytokine levels compared to untreated cells. And these increases were also not observed when DCs were pretreated with TLR4 blocking antibody (50ug/ml) (B).

**Figure S8**


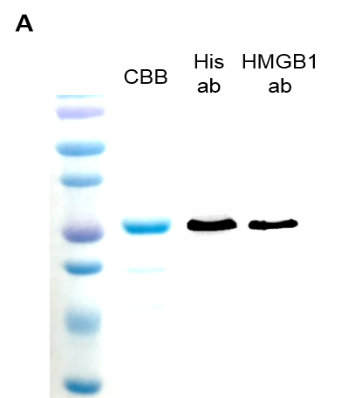

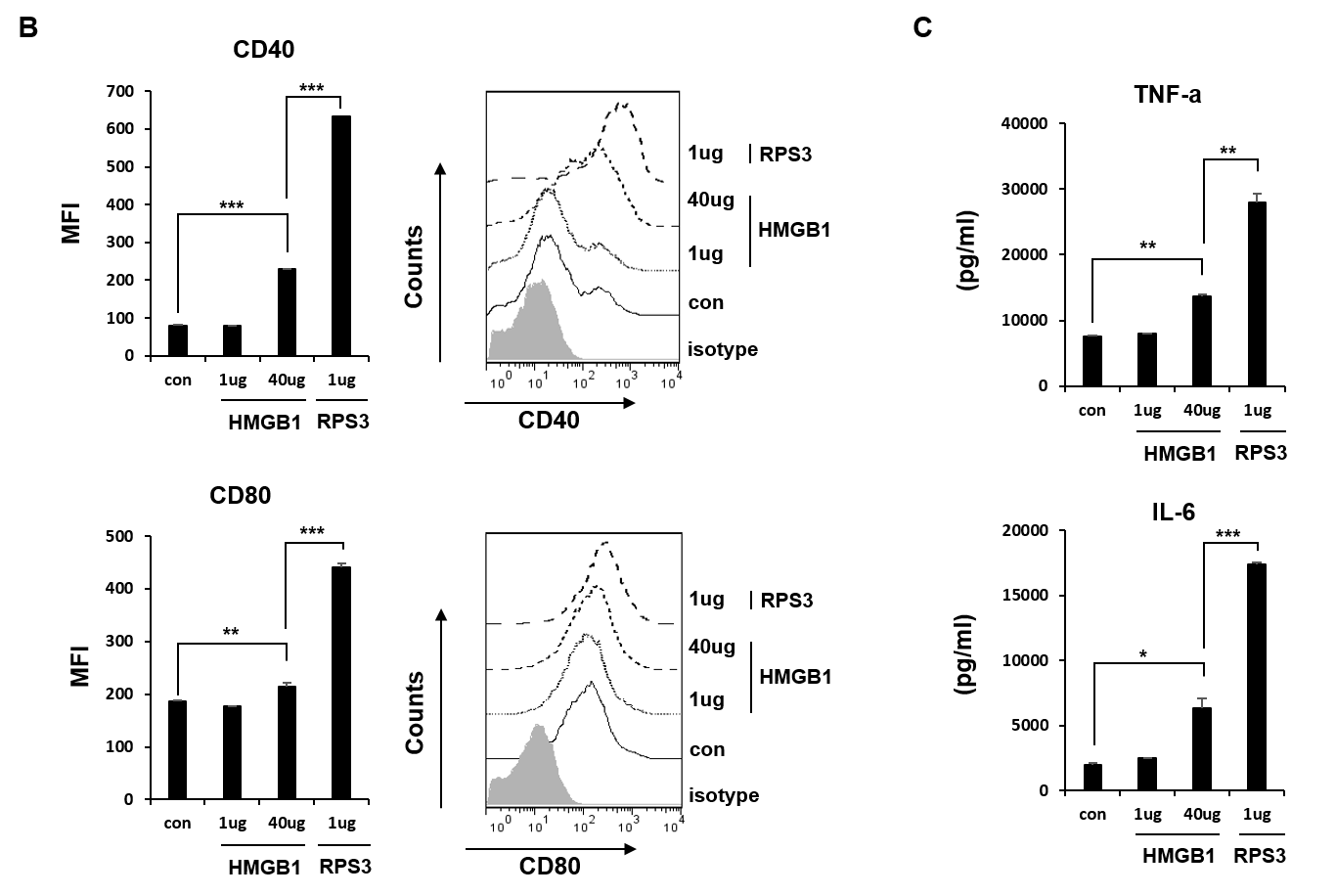


The purification of recombinant HMGB1 protein was confirmed by CBB staining and by western blot (A). The expressions of co-stimulatory molecules CD40, CD80 were increased in HMGB1 or RPS3 treated-BMDCs compared to non-treated BMDCs. Interestingly, 1ug RPS3 showed greater effects than 40ug HMGB1 (B). Next, the secretion of cytokines TNF-a, IL-6 was also increased in BMDCs treated by RPS3 or HMGB1. And the secretion levels in RPS3 treated BMDCs were higher than in HMGB1 treated BMDCs even with greatly different amounts (C).

**Figure S9**


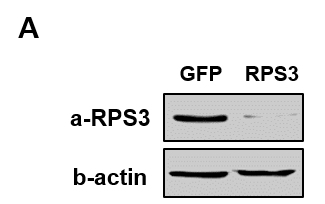


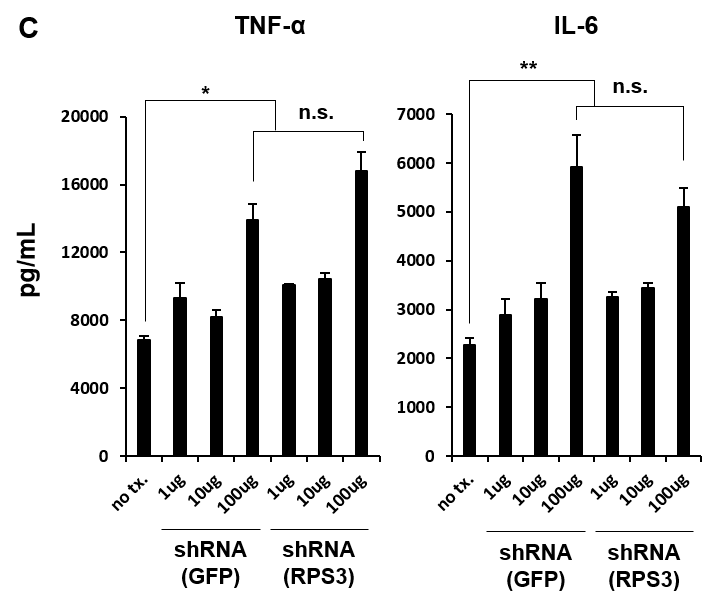

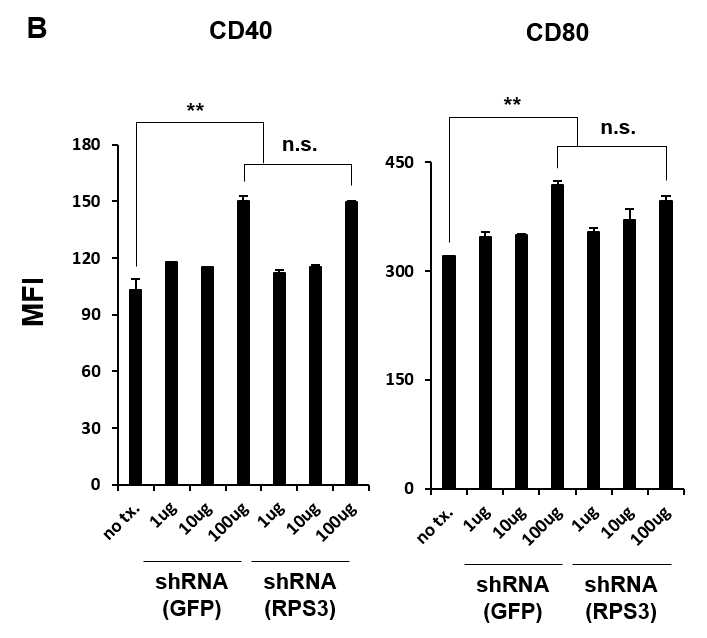


HEK293 cells were transfected by shRNA (GFP) as a negative control or shRNA (RPS3). The expression levels of RPS3 were shown by western blot (A). The lysates of HEK293 were treated on mouse BMDCs. The expression of co-stimulatory molecules CD40 and CD80 were increased by both 100ug of tumor lysates (GFP-shRNA or RPS3-shRNA) (B). The secretion of pro-inflammatory cytokines TNF-α and IL-6 was increased by both 100ug of tumor lysates (GFP-shRNA or RPS3-shRNA) (C).

**Figure S10**

**
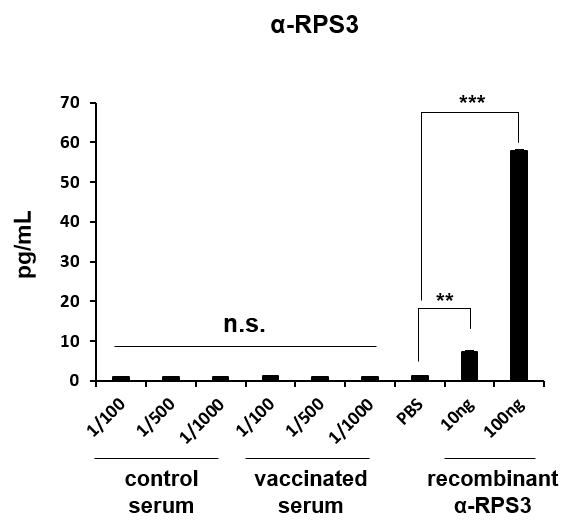
**

Mice serum with vaccination or not were used to confirm that RPS3 does not induce humoral immunity, producing autoantibodies against itself. BMDCs were treated by RPS3 and then injected into the footpads of mice once in a week for 2 times. One week after the last injection, mice were sacrificed and serum were used in ELISA. Recombinant RPS (100ng) was coated on the immunoplates. And diluted serum or recombinant RPS3 antibody were added followed HRP. As shown in the result, autoantibodies against RPS3 were not produced in neither control nor vaccinated serum. However, recombinant RPS3 antibodies as a positive control showed the increase level of RPS3.

**Figure S**


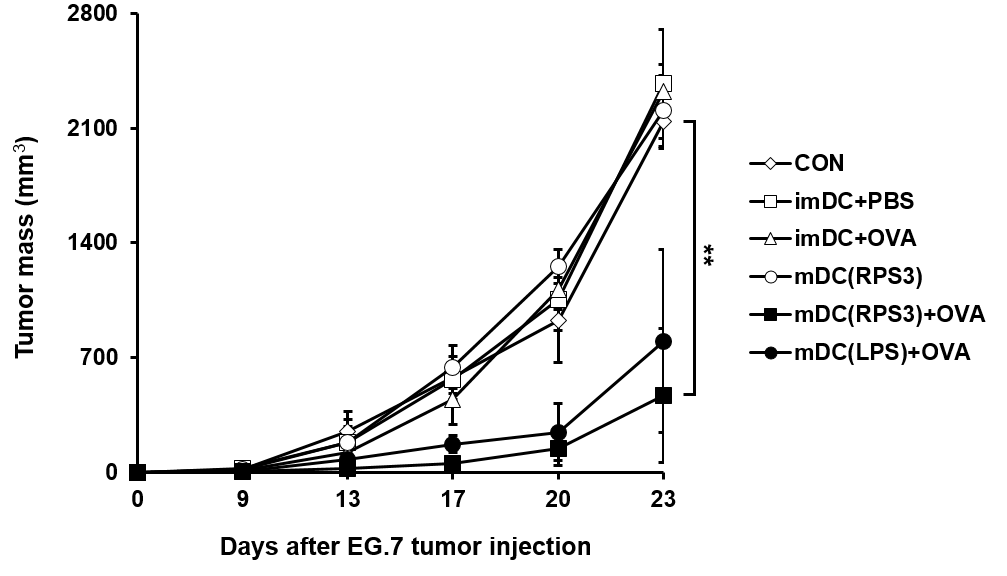


EG.7 tumor cells were injected subcutaneously into mice. Following this, mouse BMDCs were separated into the following groups: no vaccination, PBS-treated immature DCs, immature DCs loaded with tumor antigen peptide, RPS3-treated mature DCs, RPS3-treated mature DCs loaded with tumor antigen peptide, and LPS-treated mature DCs loaded with tumor antigen peptide. Five days after tumor injection, the tumor size was measured.
